# A review of starch biosynthesis in cereal crops and its potential breeding applications in rice (*Oryza Sativa* L.)

**DOI:** 10.7717/peerj.12678

**Published:** 2021-12-22

**Authors:** Ruiqing Li, Wenyin Zheng, Meng Jiang, Huali Zhang

**Affiliations:** 1 State Key Laboratory of Rice Biology and Chinese National Center for Rice Improvement, China National Rice Research Institute, Hangzhou, China; 2 College of Agronomy, Anhui Agricultural University, Hefei, China; 3 State Key Laboratory of Rice Biology, Institute of Crop Sciences, Zhejiang University, Hangzhou, China

**Keywords:** Starch biosynthesis, Endosperm, Regulator, Cereals

## Abstract

Starch provides primary storage of carbohydrates, accounting for approximately 85% of the dry weight of cereal endosperm. Cereal seeds contribute to maximum annual starch production and provide the primary food for humans and livestock worldwide. However, the growing demand for starch in food and industry and the increasing loss of arable land with urbanization emphasizes the urgency to understand starch biosynthesis and its regulation. Here, we first summarized the regulatory signaling pathways about leaf starch biosynthesis. Subsequently, we paid more attention to how transcriptional factors (TFs) systematically respond to various stimulants *via* the regulation of the enzymes during starch biosynthesis. Finally, some strategies to improve cereal yield and quality were put forward based on the previous reports. This review would collectively help to design future studies on starch biosynthesis in cereal crops.

## Introduction

With the improvement of living standards, people have higher and higher requirements for food quality. As the main component of grain crops, especially cereal crops, the content, and quality of starch will directly affect the economic benefits of crops. The harvested heterotrophic parts of staple crop plants are usually starch-storing organs such as roots (cassava, taro, and sweet potato), tubers (potato), and cereal seeds (rice, maize, wheat, barley, and sorghum) (Bahaji et al., 2014). Among of these, cereal seeds contribute to maximum annual starch production (Zeeman, Kossmann & Smith, 2010; Nuttall et al., 2017) and provide primary food to humans and livestock worldwide. However, the growing demand for starch in the food industry and the increasing loss of arable land due to urbanization emphasized the need to uncover starch biosynthesis and its regulation. The detailed regulatory mechanisms of starch biosynthesis during seed formation are largely unknown, irrespective of most starch metabolic enzymes have been identified (Thitisaksakul et al., 2012).

**Figure 1:**
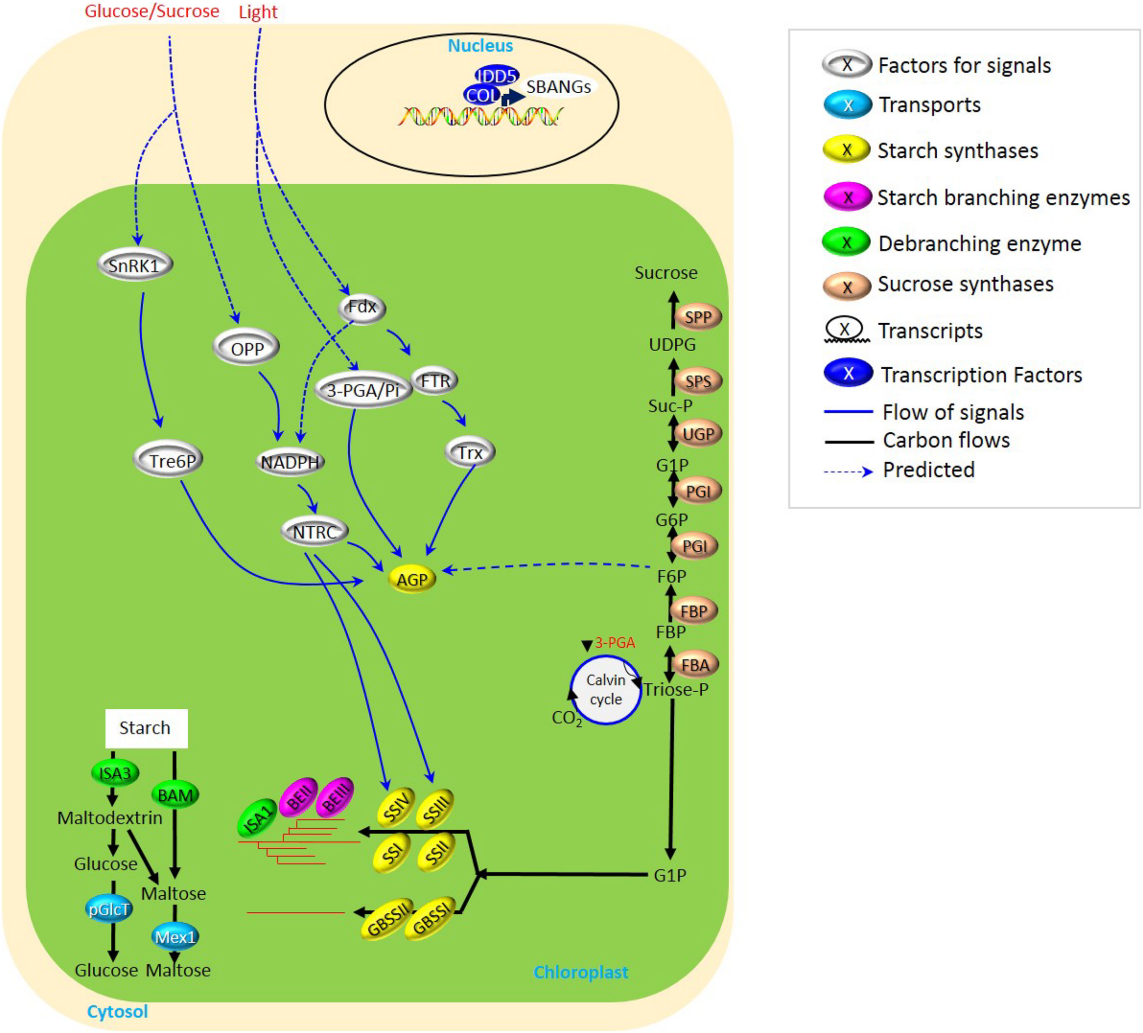
Biosynthesis of transient starch in cereal crops. Starch biosynthesis in cereal leaves displays the rhythmic turnover following the day and night oscillation of recurrent cycles of accumulation and degradation. This was largely related to the light- and glucose/sucrose-mediated signals for the regulation of starch synthases, *i.e*., AGP, SSIII, and SSIV. NADPH seemed to be served as one of the common modules of the light- and glucose/sucrose-mediated signals, while SnRK1-Tre6P performed as an independent pathway to mediate AGP. All these determine a subtle and flexible mechanism of starch biosynthesis in leaves.

Starch is composed of amylose and amylopectin glucan polymers, which are packaged to form the insoluble semi-crystalline starch granules (Sabelli & Larkins, 2009; Pfister & Zeeman, 2016). There are at least two types of synthesized starch in plants (Fig. 1). Transitory starch usually exists in the plastids of photosynthetic organs and display circadian turnover regulation with diurnal cycles (Pfister & Zeeman, 2016; Stitt & Zeeman, 2012). More importantly, the non-photosynthetic amyloplasts usually serve as the synthetic places for storage starch, which require the supply of sucrose and ATP from leaves to realize the starch synthesis (Bahaji et al., 2014). Therefore, various transporters are also essential for storage starch synthesis through the delivery of sucrose and energy by using vascular system (Geiger, 2011).

Starch synthesis requires three sorts of enzymes (Myers et al., 2000), including starch synthase (SS), branching enzymes (BEs), and debranching enzymes (DBEs). Among them, SSs are responsible for elongating glucan chains, and nowadays, six SS isoforms (SSI-SSV), granule-bound starch synthase I (GBSS/Wx) have been well characterized into the roles during starch synthesis, except for SSV (Nougué et al., 2014). SSI-SSIII are vital to establish proper amylopectin synthesis, while GBSS mainly functions in amylose synthesis (Pfister & Zeeman, 2016; Delvallé et al., 2005; Fujita et al., 2007). Besides, SSIV prefers to create branched glucans together with BEs and DBEs and to initiate granule formation (Crumpton-Taylor et al., 2013; Pfister et al., 2016; Roldán et al., 2010; Malinova et al., 2017; Lu et al., 2018). Moreover, BEs form an α-1,6-linkage *via* transferring linear glucan, and DBEs facilitate the formation of the crystalline amylopectin layers through removing α-1,6-linkages (Pfister et al., 2014; Pfister & Zeeman, 2016).

Starch accounts for 85% of the dry weight of cereal endosperm (Bahaji et al., 2014; Geigenberger, 2011) is a major storage carbohydrate formed in cereal seeds and contributes to crop yield. Starch biosynthesis in cereal crops requires coordination of starch biosynthetic enzymes and coordinates with other metabolic processes that utilize starch biosynthetic enzymes (Table S1). Thus, it is vital to engineer crops with desirable agronomic traits using biotechnological approaches (Bahaji et al., 2014) and marker-assistant breeding (Rahim et al., 2020) in crops. Based on the current references, little differences exist between several cereal crops. Nonetheless, more starch related references are reported in rice. Also, as the model plant of monocotyledonous, rice could provide referees for other species. Besides, the demand for rice quality has been getting higher and higher with the improvement of people’s living standard (Bahaji et al., 2014). The rice qualities determine its commercial value in the economic market, and have attracted the focus of both consumers and rice breeders (Bahaji et al., 2014; Geigenberger, 2011; Chen et al., 2012). With the increasing demand for rice of good qualities, it is vital to explore starch biosynthesis and its regulatory mechanisms, which would be important for the oriented genetic improvement of rice qualities.

Nowadays, there are many published reviews about starch biosynthesis in plants (Geigenberger, 2011; Bahaji et al., 2014; López-González et al., 2019). Based on the timeliness of starch research, more recent works on starch biosynthesis have been focused on the transcriptional factors and the regulatory mechanisms. This review furthermore updated the works of starch metabolism in cereal crops on the basis of previous reviews. Since grain development is limited by the duration of flag leaf photosynthesis (Borrill et al., 2015) and associated with the sugar levels and activities of important starch-synthesizing enzymes (Fahy et al., 2018) during seed development in cereal crops, we initiated to summarize the regulatory signals in leaf starch biosynthesis. Subsequently, we paid more attention to how transcriptional factors (TFs) systematically respond to various stimulates *via* regulations of the enzymes during endosperm starch synthesis. Finally, the molecular mechanism of starch synthesis was summarized and strategies for rice yield and quality improvement were discussed, providing theoretical basis for improvement and breeding in rice. Some strategies were put forward to improve cereal yield and quality based on the previous reports. Our review provides a critical review of the studies on starch biosynthesis regulation and some potential starch-related strategies for applications in crops.

## Survey Methodology

To complete this article, an electronic literature search was performed exhaustively on the databases of Web of Science, Google Scholar, Science Direct, Mendeley and EndNote using key words such as “starch metabolism”, “cereal crops”, “transcription factors”, “transient starch”, “endosperm starch”, “rice”, “maize”, “wheat”, “Barley”, “sucrose-to-starch”, integrated with the usage of “+”, “or”, “AND” for specific search returns. Works of the past 20 years (up until Aug 29^th^, 2021) were mostly focused here. More than eight hundred publications were retrieved, and article selection was conducted according to previous method (Moher et al., 2009). First of all, the duplicated articles were deleted. Subsequently, unrelated articles were then screened out after examining the titles and abstracts. Eventually, the most relevant articles in English were used to complete this review.

### Biosynthesis of transient starch in cereal crops

Compared to many studies on the biosynthesis of transient starch in other plants, *i.e*., *Arabidopsis*, few have focused on cereal crops. This is largely attributed to the phenotypical obscurity derived from the aberrant metabolism of transient starch in cereal leaves and the inedible traits of leaves. However, although sucrose-to-starch metabolism occurs with transport *via* phloem to sink tissues (Macneill et al., 2017), due to utilizing fructose phosphates to glucose phosphates to ADP-glucose, transient starch metabolism in leaves is vital for the formation of seeds, and nowadays, it is also helpful to explore the source of potential biofuels to relieve energy shortage. Several reviews (Zeeman et al., 2002; Geigenberger, 2011; Stitt & Zeeman, 2012) have focused on starch metabolism in plants. Our review highlights the possible signaling of starch biosynthesis explicitly upon starch metabolism under light/darkness alternation in crops, including glucose/sucrose signals (Fig. 1 and Table S1).

#### Light-dependent signaling pathway

The rhythmic turnover following the day and night oscillation of recurrent cycles of accumulation and degradation has been displayed through the biosynthesis of transient starch in cereal leaves (Bahaji et al., 2014; Fernandez et al., 2017). However, various types of light/darkness alternation and glucose/sucrose availability, as well as protein-protein interaction, determine a subtle and flexible mechanism of starch biosynthesis in leaves (Fig. 1 and Table S1).

Because light-dependent photosynthesis provides raw materials for starch synthesis, the synthetic rate of starch in leaves is regulated to encounter the fluctuating day length. However, fluctuation of day length usually temporarily generates a period of carbon shortage, which is mainly dependent on the light-dependent rebalance of the carbon budget. The rebalance of the carbon budget is usually realized by accelerating the starch synthesis while hindering the rate of starch degradation (Graf & Smith, 2011). Moreover, from a part of the newly fixed carbon during the light period, starch was synthesized and then degraded into glucan in the following night period, termed as starch turnover (Bowsher et al., 2007; Lee et al., 2016). This promotes starch turnover to synchronize with the day length (Fernandez et al., 2017; Davies et al., 2003; Pan, Strelow & Nelson, 1990), which maximizes the efficiency of carbon use. Therefore, starch turnover is essential for plant development and biomass generation, and more importantly, is an effective and efficient response to light-dependent environment adaptation.

Light-dependent signaling usually realizes its regulation to starch synthesis through the control of starch biosynthetic enzymes. ADP-glucose pyrophosphorylase (AGPase) was supposed to be one of the most studied enzymes involved in the light-dependent signaling pathway, in which the regulation of AGPase was largely related to the modules of photosynthesis. For example, ferredoxin (Fdx), thioredoxins (Trx), and Fdx-dependent Trx reductase (FTR) participated in the regulation of AGPase in response to light (Lunn et al., 2014; Davies et al., 2003). Besides, through nicotinamide–adenine dinucleotide phosphate (NADPH) and NADP-thioredoxin reductase (NTRC), light-induced Fdx also regulated the expression of AGPase/starch synthase III (SSIII)/SSIV/β-amylase (BAM) (Yadav et al., 2014). 3-phosphoglycerate (3-PGA; Guo et al., 2012) and fructose-6-phosphate (F6P; Koumoto et al., 2013) were also involved in the regulation of AGPase, but the detailed mechanisms were not very clear. Collectively, for transient starch biosynthesis, light is vital to regulate the activities of various starch biosynthetic enzymes through (i) 3-PGA→AGPase, (ii) Fdx→FTR→Trx→AGPase, and (iii) Fdx→NADPH→NTRC→AGPase/SSIII/SSIV/BAM (Fig. 1).

#### Glucose/sucrose-dependent signaling pathway

**Figure 2:**
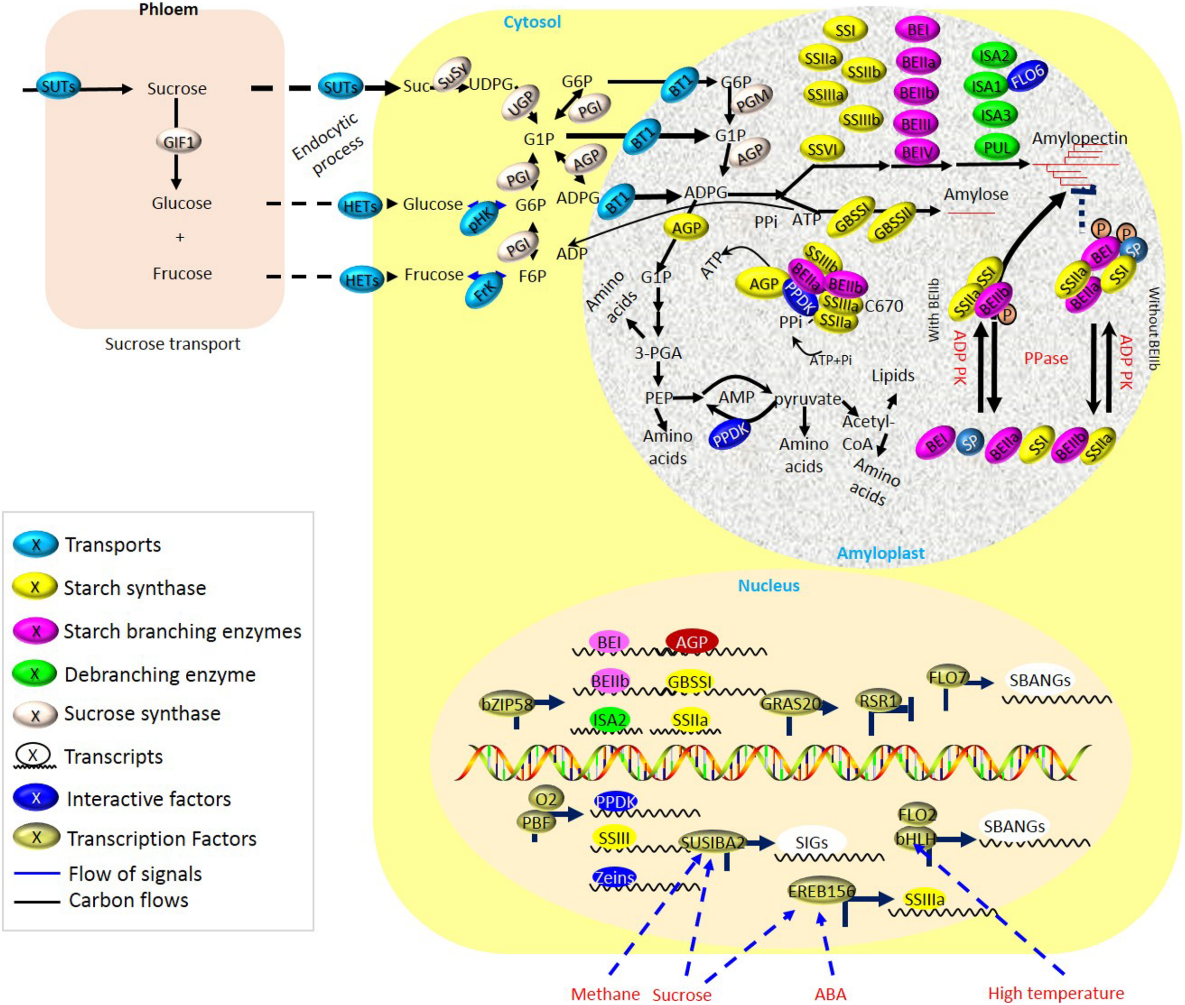
Biosynthesis of storage starch in cereal crops. During the grain filling period, sucrose localized in vegetative organs (*i.e*., leaves) is intensively unloaded from the phloem and transported into the reproductive organs (*i.e*., grains). Multiple starch biosynthetic enzymes co-operate to produce starch granules both in transient and storage starch biosynthesis. Grain-filling is associated with the normal development of amyloplasts, delivery of carbon precursors and energy from leaves to endosperm by sucrose transporters (SUTs), and enzymes of endosperm starch biosynthesis during grain-filling periods, which are greatly susceptible to fluctuated environments.

As important forms of carbon budget, glucose and sucrose also function in the regulation of carbon allocation during starch biosynthesis. Nonetheless, this is a very complex process. More than that, glucose and sucrose are also greatly related to the balancing of growth and reproduction using the available carbon, which involves trehalose 6-phosphate (Tre6P) in higher plants (Fig. 2).

Tre6P, an intermediate of trehalose (TRE) biosynthesis, functions in the sucrose-Tre6P sensor nexus (Paul et al., 2010; Martins et al., 2013). It involves the coordination of carbon and nitrogen metabolism in plants (Bledsoe et al., 2017; Paul et al., 2008). To optimize the intracellular sucrose concentrations for plants’ growth and development, Tre6P usually acts as both a signal and a negatively retrograde regulator (Yadav et al., 2014). Although the contents of Tre6P and TRE are low, Tre6P is essential in making sucrose available to organs, correlating strongly with changes in available carbon (Martínez-Barajas et al., 2011). During sucrose and starch metabolism, the interaction of Tre6P with the sucrose nonfermenting1-related kinase 1 (SnRK1) system occurred (Zhang et al., 2009; Nunes et al., 2013). It provided new views on the allocation of carbon to the developing cereal grains.

In fact, in the endosperm of cereal crops, the deposition of starch grains is closely connected with Tre6P. In several cereal crops, a model for the role of Tre6P in carbon partitioning and yield was proposed and confirmed (Langlois, Shulman & Arbesman, 2015). Local sucrose availability played key roles in Tre6P/SnRK1 signaling to determine yield and grain quality (Martínez-Barajas et al., 2011; Lawlor & Paul, 2014). In Tre6P/SnRK1 signaling, another key factor was trehalose-6-phosphate phosphatase 7 (TPP7), which increased Tre6P turnover and served as an energy sensor (Kretzschmar et al., 2015). Also, TPP7 could measure anabolism or catabolism depending on the availability of local sucrose (Nuccio et al., 2015), thereby enhancing starch mobilization to trigger the growth dynamics of the germinated embryo and elongated coleoptile sheath. This consequently improved anaerobic germination tolerance in direct-seeded rice (Kretzschmar et al., 2015). Because of high sucrose concentration, the accumulation of Tre6P inhibited SnRK1 to promote growth. However, low sugar promoted the conversion from Tre6P to TRE, and therefore, phosphorylated SnRK1 to activate C/S1 basic leucine zipper (bZIP) transcription factors (TFs) for resource sequestration in sinks (Langlois, Shulman & Arbesman, 2015). The Tre6P-C/S1 bZIP-SnRK1 regulatory module existed in both monocotyledon (*i.e*., rice; Cho et al., 2012) and dicotyledons (*i.e*., *Arabidopsis*; Lunn et al., 2014; ƠHara, Paul & Wingler, 2013). The effective nutrient-sensing system served as a spatial-temporal regulator (Langlois, Shulman & Arbesman, 2015; Cho et al., 2012), which improved the yield stability of staple crops under adverse conditions. Aside from light, sucrose or glucose also served as another factor in activating the signaling of NADPH-NTRC to regulate AGPase/SSIII/SSIV (Guo et al., 2012). Nonetheless, oxidative pentose phosphate (OPP) and SnRK1 (Nunes et al., 2013) were supposed to be the first sensors for sugar.

Collectively, the accumulation of sucrose was vital for plant growth through regulation of starch biosynthesis, and several sucrose-dependent signaling has been summarized here, including (i) sucrose→SnRK1→Tre6P→AGPase and (ii) sucrose→OPP→NADPH→NTRC→AGPase/SSIII/SSIV (Fig. 1).

#### Circadian rhythms for starch biosynthesis

Circadian rhythm is also an important regulator for starch biosynthesis. Starch biosynthesis followed the circadian rhythms (Annunziata et al., 2017) and relied on the duration and light intensity (Fernandez et al., 2017). Thus, based on the light-dependent activity of starch-degrading enzymes, starch was more susceptible to degradation. This was partly attributed to the transcript levels of *β*-amylase 3 (BAM3), the major starch degrading enzyme with a short half-life (Li et al., 2017a). However, many other starch degradation-related enzymes, including glucan water dikinase (GWD), *α*-amylase 3 (AMY3), and disproportionating enzyme 2 (DPE2), had long half-lives over the day/night cycle (Skeffington et al., 2014; Baerenfaller et al., 2012; Nelson et al., 2014; Wu et al., 2002). This also determined that their activities were probably not regulated at the gene expression level. For example, to activate enzymes’ activities and initiate starch breakdown in light, the transcription levels of AMY3- and BAM1-encoded genes were greatly upregulated in the present osmotic stress. Nonetheless, these two enzymes were not essential for diel starch metabolism without abiotic stress conditions (Fulton et al., 2008; Horrer et al., 2016; Thalmann et al., 2016). Inversely, in the presence of light, a higher increased propensity of starch degradation with time largely depended on phosphorylation of BAM1 and AMY3 (Fernandez et al., 2017; Thalmann et al., 2016). The phosphorylation regulation was achieved by adding phosphate groups to glucose (Glc) residues with two GWDs and phosphoglucan water dikinase (PWD). This reduced the level of crystalline organization of the granule matrix and exposed the surface of BAMs to attack, thus causing an increased propensity for starch degradation with time during the light period (Scialdone et al., 2013; Fernandez et al., 2017). Therefore, the elaborate regulation of enzymatic activities is tightly related to environmental conditions.

Besides the light period, several starch biosynthetic enzymes were prone to function in dark conditions. For example, GBSS/Wx elongated the amylose polymers released from the starch granule and rapid degradation of starch granule at night (Ortiz-Marchena et al., 2014). Another was early starvation 1 (ESV1), a relatively novel starch degradation factor, which was essential for controlling the starch breakdown rate at night (Feike et al., 2016). Nonetheless, through phosphorylation/dephosphorylation enzymes, ESV1 performed a distinguished working mode and directly mediated starch-bound phosphate (Feike et al., 2016). Besides, for starch degradation, ESV1 and its homologs also displayed spatiotemporal specificity. In the absence of ESV1, for promoting the accumulation of maltose from starch degradation, starch granules in leaves appeared to be accessible to hydrolytic enzymes during the day and night (Weise, Weber & Sharkey, 2004). However, starch storage may be prevented in other organs due to simultaneous biosynthesis and starch degradation (Feike et al., 2016).

The phenomenon of light alternating between day and night also made the metabolism of starch rhythmicity. It was mainly realized through the starch-degrading enzyme (Fig. 1). Compared with the regulation of starch synthase at the gene expression level, phosphorylation regulation of long half-life enzyme activities appeared to be more conducive to the biological adaptation of light rhythm and biological stress. Besides, some of the regulatory elements and enzymes displayed certain temporal and spatial expression specificity in the biosynthesis of starch. Therefore, there has been a certain difference between the synthesis of temporary starch and the synthesis of storage starch. It might help realize the effective regulation of seed formation to bring clarity in the commonness and difference.

### Delivery of the carbon precursors from leaves to developing grains

For energy production and starch biosynthesis, sucrose synthesized in leaves must be transported from the leaves throughout a long-distance vascular pathway (phloem) to the developing grains (Fig. 2 and Table S1). Three types of sucrose transporters (SUTs or SUCs) have been identified in plants (Reinders, Sivitz & Ward, 2012; Kühn & Grof, 2010). Multiple SUTs have now been identified in cereal crops, including C3 grasses such as rice (Sun et al., 2010), wheat (Aoki et al., 2004), and barley (Haupt et al., 2001), and C4 grasses, including maize (Baker et al., 2016; Guo et al., 2012) and sweet sorghum (Bihmidine et al., 2016). The type I SUTs are unique to eudicots and play roles in loading and retrieval of Suc in the transport phloem (Gould et al., 2012). For type II SUTs, at least three functions have been proposed in cereals (Aoki et al., 2003; Scofield et al., 2002, 2007; Sauer, 2007; Baker et al., 2016), including (i) Suc phloem loading in leaves, (ii) phloem unloading of Suc in sink tissues, and (iii) retrieval of leaked Suc. Among of them, the type II SUTs in rice has been showed the likewise functions (Ishimaru et al., 2001; Scofield et al., 2002, 2007). Besides, the type III SUTs localizes at vacuolar membrane and functions in sucrose-uptake (Schulz et al., 2011). Besides, at least one Type III SUT exists in each plant species. By regulating the energy status and controlling flowering, sucrose supply to the filling grain was crucial for crop yield and quality (Kühn & Grof, 2010). SUTs were tightly regulated depending on fluctuating environments such as light and photoperiod, and internal stimuli such as phosphate (P) starvation, sucrose leakage, and H^+^ leak (Fig. 2).

Sucrose transporters appear to be relatively conserved within the *Viridiplantae* plants, despite limited studies on the regulation of SUTs in cereal crops. The identification of sucrose transporters in cereal crops could be aided by the complete genome sequences of Arabidopsis and potato.

### Biosynthesis of storage starch in cereal crops

Aside from sucrose and ATP from leaves, normal development of differential plastids, amyloplasts, are essential for the synthesis of endosperm starch, and thereby, there are some common traits existing between chloroplasts and amyloplasts. During starch biosynthesis, expression of starch biosynthetic genes was proposed to be regulated by tetrapyrrole intermediates both in BY-2 cultured cells (Enami et al., 2011) and in rice (Li et al., 2021). The mutation of rice *genome uncoupled 4*, which was revealed as one predominant regulator of chlorophyll biosynthesis, performed negative effects on the starch synthetic genes, such as *GBSSI* and *AGPS1*, in endosperm during early seed development, partially through mediating the accumulation of heme (Li et al., 2021). Obviously, the well-functioning of starch biosynthetic enzymes also plays key roles during starch synthesis (Fig. 2), whereas mutations of the genes encoding starch biosynthetic enzymes usually lead to negative yield and/or quality of crops. Nonetheless, part of the mutation also contributes to some peculiar features of starchy endosperm, which could serve as excellent features in breeding applications (*i.e*., floury; Table S1). Obviously, one complex regulatory network seems to be employed for the accumulation of storage substances and relies on coordination among different metabolic and cellular processes.

#### Regulators of transcriptional factors

**Figure 3:**
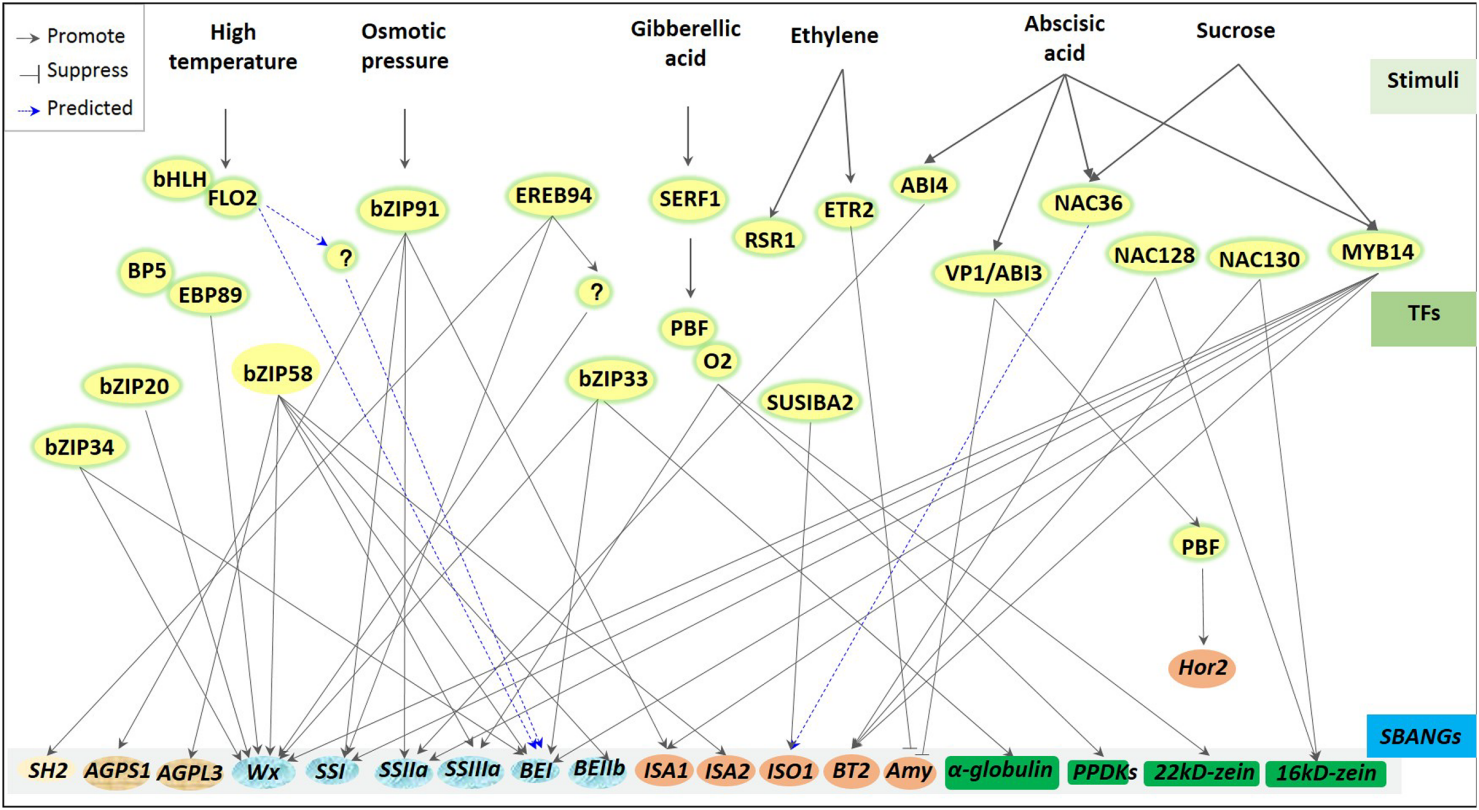
Regulators of transcriptional factors involved in starch biosynthesis of cereal crops. A great deal transcription factors (TFs), including bZIPs, bHLHs, NACs and MYBs, are involved in the regulation of starch biosynthesis, and functions with the different conditions, especially under the environmental stress (*i.e*., high temperature, osmotic stress) and the stimulations of various phytohormones, including ABA, GA, and ethylene. Response of TFs to the stimulates usually showed systematic transcriptional regulation. Almost all TFs control multiple genes, including bZIP58, MYB14, bZIP91 and so on; in turn, one gene may also response to several TFs, *e.g*., *Wx* (*GBSSI*), *BEI* et al. All these suggests that one complex and elaborate regulated network is existed during starch synthesis in cereal crops, despite showing many unknown mechanisms.

Regulation of transcriptional factors for storage starch biosynthesis has been greatly reported in plants (Mangelsen et al., 2010; Tiessen et al., 2012; Fu & Xue, 2010) and greatly depends on varied environments (Fig. 3). For example, high temperature stress usually downregulated the expression of starch synthesis-related genes, including GBSS, branching enzyme I (BEI; Blauth et al., 2002; Satoh et al., 2003), substandard starch grain (Matsushima et al., 2014; 2016), and BEIIb (Nishi et al., 2001; Regina et al., 2005), whereas ABA and sucrose induced the ZmSSIIIa expression (Huang et al., 2016). Although diverse starch biosynthetic genes were subjected to be driven through different specific TFs, some regulations appeared to have pleiotropisms or multigenic effects. One ZmEREB156 specifically combined with ZmSSIIIa promoter to regulate starch synthesis in maize (Huang et al., 2016). However, to regulate starch synthesis in rice endosperm, one leucine zipper bZIP58 showed wide compatibility to regulate several genes (Zhang et al., 2012; Hanashiro et al., 2008; Lee et al., 2007; Wei et al., 2017), including *OsAGPL3*, *OsGBSSI* (*Wx*), *OsSSIIa*, *BEI*, *OsBEIIb*, and isoamylase-type debranching enzyme 2 (*ISA2)*. Besides, *GBSSI* was reported to be regulated by several TFs, including bZIP33, bZIP34 (Wang et al., 2013), and bZIP58 (Onodera et al., 2001) in rice and ZmMYB14 (Xiao et al., 2017) and prolamin-box binding factor/opaque 2 (PBF/O2) (Zhang et al., 2016a) in maize. Nowadays, many TFs involved in endosperm starch biosynthesis have been reported, including GRAS20 (Cai et al., 2017), ethylene response 2 (ETR2; Wuriyanghan et al., 2009), bZIP91 (Zhang et al., 2016b), NAC36 (Chen et al., 2016), FLOURY ENDOSPERM7 (FLO7) (Zhang et al., 2014; Li et al., 2014a), and SUSIBA2 (Sun et al., 2003; Su et al., 2015) in rice, barley, and maize. However, most of them have not been explored in specific regulated genes with their given degree of studies.

Besides, transcriptional regulation on starch biosynthesis can also be realized through TF combination with other factors. Under high temperatures, starch biosynthesis-associated nuclear genes (SBANGs) were regulated by the interaction of FLO2 and bHLH (She et al., 2010). One novel FLO2-interacting protein was demonstrated to maintain fertility and seed quality in rice (Suzuki et al., 2020). Regulation of pyruvate orthophosphate dikinase (PPDK), SSIII, and zein during protein and starch synthesis were subjected to the co-interaction of O2 and PBF (Zhang et al., 2016a).

Additionally, in different cereal crops, transcriptional regulation on starch biosynthesis also showed a certain degree of conservation. The DOF transcription factors bound to GaMyb to mediate α-amylase (AMY) in rice (Yamamoto et al., 2006; Kawakatsu et al., 2009) and in barley (Diaz et al., 2002), while a WRKY transcription factor SUSIBA2 regulated endosperm starch synthesis in barley and rice (Sun et al., 2003; Su et al., 2015). Moreover, the bZIP transcription factor was also bound to a conserved cis-element to regulate storage starch and storage proteins in maize (Zhang et al., 2016a), wheat (Albani et al., 1997), and rice (Onodera et al., 2001). Recently, one endosperm-specific NAC-type TF, TaNAC019, was revealed to directly mediate the accumulation of storage proteins *via* TaSPA and TaGAMyb to regulate SSIIa and sucrose synthase 1 (SuSy1) consequently affecting the starch accumulation in wheat (Gao et al., 2021). This was distinguished with previous studies of ZmNAC128 and ZmNAC130 that regulated the transcription of brittle 2 (BT2) and 16-kDa *γ*-zein in maize (Zhang et al., 2019). Still, both were subjected to the regulation of the balance between storage starch and storage proteins. Interestingly, the balance between storage starch and storage proteins also required spatio-temporal coordinating TFs and was subjected to the control of other pivotal upstream regulators to these TFs. In maize, during seed filling, a group of spatio-temporal coordinating transcription factors (including OPAQUE2, Prolamin Binding Factor1, NAC128, NAC130, and OPAQUE11) were reported to regulate the synthesis of starch and protein in endosperm. And ZMABI19 bind to the promoters of O2 and other transcription factors (*i.e*., Prolamin Binding Factor1, NAC128, NAC130, and OPAQUE11) that play a key role in seed filling and plant hormone response to regulate their expression (Yang et al., 2020). However, the pivotal role of ZMABI19 in maize seed development still needs to be further clarified by exploring the regulation mechanism of ZMABI19 at both transcriptional and translational levels, as well as other upstream factors regulating embryo and endosperm development in coordination with ZMABI19 (Yang et al., 2020). To sum up, the regulation of transcription factors on starch biosynthesis shows different levels and forms in diverse environments (Fig. 3).

#### Functions of starch biosynthetic enzymes

The function of starch biosynthetic enzymes usually depends on posttranslational regulation, including phosphorylation, the formation of a transient complex, and so on (Fig. 2). For starch biosynthesis in cereal endosperms, the phosphorylation-dependent multienzyme complex is essential. The phosphorylation-dependent SSI/SSIIa/BEIIa/BEIIb complex was detected in barley endosperm (Ahmed et al., 2015; Hirose & Terao, 2004). From SSIIa/SSIIIa/SSIVb/BEI/BEIIb/pullulanase (PUL) in the amyloplast of rice endosperm, a 700 kD protein complex could be formed (Crofts, Nakamura & Fujita, 2017; Guo et al., 2017; Fujita et al., 2009). The formation of BEs and SSs complexes depends on the phosphorylated BE and phosphorylase 1 (Pho1) isoenzymes, while their segregation is activated upon the suppressed dephosphorylation (Pang et al., 2018; Rahman et al., 1997; Secco, Baumann & Poirier, 2010; Kang et al., 2013; Mizuno et al., 2001). Besides, the regulation of phosphorylation is also greatly associated with the seed development period. A 260 kDa SSI/BEIIa/BEIIb complex formed in the middle and late development period of the grain endosperm instead of the early stage (Tetlow et al., 2008). However, the SSIIa/SSI/BEIIb tripolyprotein complex of the initial amyloid matrix participated in synthesizing a starch-branched chain during late seed development (Liu et al., 2014, 2012a). Interestingly, the formation and action sites of the starch complex were also different. The phosphorylation site of SSIIa and BEIIb varied with species (Crofts, Nakamura & Fujita, 2017; Li & Gilbert, 2016). For example, the amino acid sequence of Thr323 was conserved in rice, maize, and barley but not in wheat. A 670 kDa protein complex consisting of PPDK/AGPase/SSIII/SSIIa/BEIIa/BEIIa was formed to regulate carbon distribution among amino acids, lipids, and starch in the maize endosperm (Guo et al., 2012). The Ser residue of BEIIb was phosphorylated by kinases, of which Ser286 and Ser297 were highly conservative between species, but the phosphor-Ser649 was not conservative, which seemed to be confined to the enzyme in cereals and was not universal (Makhmoudova et al., 2014; Liu et al., 2012).

Importantly, as an important rate-limiting enzyme for starch synthesis, AGPase subunits form a complex with different roles of large and small subunits to make ADP-Glucose, and thereby, the enhanced activity of AGPase would greatly increase the starch content (Geigenberger, 2011). AGPase was activated by sucrose (Tiessen et al., 2012), pyridoxal, DTT, and 3-PGA, while suppressed by Pi and nitrate (Scheible et al., 1997) to adapt to environmental changes (Geigenberger, 2011).

Starch biosynthetic enzymes also functioned *via* the formation and segregation of transient complexes (Fig. 2). Proteomic analysis of soluble fractions from different developmental stages of seeds or endosperms showed that the upregulated expression of many starch-biosynthetic enzymes led to efficient starch biosynthesis in different cereal crops (Mechin et al., 2007; Xu et al., 2010; Mu et al., 2009; Kazuaki, Kanako & Kazufumi, 2010; Long et al., 2017; Satoh et al., 2008). It was also well demonstrated that the size of starch granules correlated with the activity of granule-associated starch biosynthetic enzymes in wheat (Cao et al., 2015). Likewise, in maize endosperms, the formation of starch granules were associated with phosphorylation modification of the transient complexes that constituted GBSS, SSI, SSIII, BEI, BEIIa, BEIIb, and Pho1 (Grimaud et al., 2008). The developmentally altered SGAPs mainly played significant roles in polyglucan elongation and granule structure modification in developing rice endosperms (Yu & Wang, 2016).

Here, to promote efficient carbon partitioning during starch and protein storage, a synergetic network is composed of starch biosynthesis and protein biosynthesis, as well as protein folding and PPDK pathways (Fig. 2 and Table S1).

#### Granule formation in starch biosynthesis

Granule formation in storage starch shows distinct specific mechanisms from transient starch, and some related enzymes, including SSIV, FLO6 and ISA (Fig. 2). SSI, SSII, SSIII, and GBSS mainly showed activity on linear oligosaccharides, while SSIV seemed to focus on linear maltooligosaccharides (Ryoo et al., 2007; Cuesta-Seijo et al., 2016; Lu et al., 2018; Dian, Jiang & Wu, 2005; Fujita et al., 2006). SSI is referred to as “DP < 10, soluble starch synthase” with an assigned role of type of amylopectin structure, when the soluble starch synthases extends progressively longer glucan chains from SSI to SSIII, with SSIV plays roles in granule initiation of starch (Kosar-Hashemi et al., 2007; Fujita et al., 2011; Cuesta-Seijo et al., 2016).

Moreover, FLO6 serves a vital function to regulate starch biosynthesis and granule initiation of endosperm starch in rice (Peng et al., 2014). Unlike in *Arabidopsis* leaves, the initiation of starch granules in developing seeds largely depends on the distinct interaction with ISA1 in rice, *e.g*., the interaction of FLO6 with ISA1 (Dinges et al., 2003; Utsumi et al., 2011; Peng et al., 2014). Interestingly, PROTEIN TARGETING TO STARCH 2 (PTST2), the homologous protein of FLO6 in leaves, is not interacted with ISA1, whereas the interaction between PTST2 and SSIV is explored to affect granule numbers (Seung et al., 2017). Nonetheless, PTST2/FLO6 performs a conserved function in granule initiation of leaf and endosperms, but further investigations are required to determine the specific mechanism.

### Starch synthesis in rice

As one of the most important food crops, grains of rice (*Oryza sativa* L.) consist of embryo, endosperm and seed coat, among which endosperm is a major storage organ for grain development (Yu & Wang, 2016). As the major storage material, starch accounts for 85% dry weights of the cereal endosperm, and thus, serves as one main food source for human beings (Ferreira et al., 2017). Since utilizations of the dwarf gene (Miura et al., 2009) and heterosis (Tian et al., 2009), the yields of rice have been significantly improved twice. However, with the improvement of people’s living standard, the demand for rice quality has been getting higher and higher. Therefore, the rice qualities, especially for eating and cooking qualities (ECQs), determine its economic value and consumer recognition in the commercial market, and have attracted the focus of both consumers and rice breeders (Lau et al., 2015). Rice quality mainly includes appearance characteristics, ECQs and minor element contents (Rabiei et al., 2004; Okpala et al., 2017). However, rice quality shows various preferences due to differences in dietary culture (Ferrero & Nguyen, 2004). Although some regions prefer trace elements and appearances (Lau et al., 2015), more regions, including China and some European countries, pay more attention to taste qualities (Ferrero & Nguyen, 2004). Therefore, starch not only is the main storage form of carbohydrate in plants, but also performed important values of biological and economical (Sulpice et al., 2009; Zeeman, Kossmann & Smith, 2010). With the increasing demand for rice of good qualities, it is vital to explore starch biosynthesis and its regulatory mechanisms, which would be important for the oriented genetic improvement of rice qualities.

As shown in above, starch deposition of grains depends greatly on T6P in endosperm (Lawlor & Paul, 2014; Yadav et al., 2014). In rice, OsTPP7 is involved in increased Tre6P turnover and plays central roles to promote starch mobilization (Kretzschmar et al., 2015). A model has been proposed to illuminate the roles of T6P in carbon partitioning and plant yield (Langlois, Shulman & Arbesman, 2015). Accumulation of T6P induced by high sucrose inhibits the SnRK1 activity to repress growth, while low sugar levels would promote the conversion of TRE from T6P, and thereby, the phosphorylated SnRK1 subsequently activate C/S1 bZIP transcription factors for resource sequestration in sinks (Langlois, Shulman & Arbesman, 2015). This model is confirmed by several reports from cereal crops. The allogeneic expression of the *OsTPP1* gene in developing maize ears significantly improved yield stability under both normal conditions and mild drought stress (Nuccio et al., 2015). Also, in OsMADS6-TPP1 maize transgenics, lower T6P contents but increased sucrose levels were observed in ear spikelets and developing ears, indicating the function of MADS in the improved sink of reproductive tissues (Nuccio et al., 2015). Interestingly, the T6P-C/S1 bZIP-SnRK1 regulatory module has been revealed in *Arabidopsis* (Ma et al., 2011; ƠHara, Paul & Wingler, 2013; Lunn et al., 2014). Interestingly, Tre6P not only plays roles in the sucrose-Tre6P sensor nexus (Martins et al., 2013) but also involves the coordination of carbon and nitrogen balance (Figueroa et al., 2016). Using ^14^CO_2_ and ^13^CO_2_ labelling, the increased Tre6P has been revealed to decrease the sucrose content, but increase the levels of amino acids (Figueroa et al., 2016).

The mutation of *OsFLO6*, an ortholog of Arabidopsis PROTEIN TARGETING TO STARCH 2 (PTST2), affects the endosperm starch synthesis and alters starch properties of, which is associated with defects in granule initiation (Peng et al., 2014; Seung et al., 2017). However, mechanism underlying starch granule initiation in rice grains maybe different from *Arabidopsis* leaves (Peng et al., 2014). Rice FLO6 may interact with ISA1 based on protein interaction assays (Peng et al., 2014), while no evidence indicates *in vivo* interaction of PTST2 with ISA1 in *Arabidopsis* by using both *isa1* and *ptst2* mutants (Seung et al., 2017; Delatte et al., 2005). Unlike as an ISA1/ISA2 complex in Arabidopsis, ISA1 primarily exists as a homodimer in rice (Streb & Zeeman, 2012). Aside from possessing starchless amyloplasts, the ISA1-deficient mutants in rice also failed to initiate starch granule, interestingly, the both traits were presented in *flo6*, but the phenomenon of accumulate phytoglycogen as shown *isa1* was not detected in *flo6* (Kawagoe et al., 2005; Peng et al., 2014). Therefore, granule initiation in cereals endosperm requires the conserved PTST2/FLO6 complex as that in leaves (Peng et al., 2014; Seung et al., 2017), but its underlying mechanisms still requires more studies, especially for different plant tissues.

### Strategies for grain improvement through starch biosynthesis

Compared with conventional breeding, genetic engineering seems to be more cost-effective and efficient to improve the screening of grain quality. Biosynthesis of storage starch requires the transport of carbon precursors and ATP from leaf organs to storage organs such as developing seeds through a long-distance transport of phloem. Further transformation of sucrose starch into amyloplasts through a series of enzymatic activity reactions and the transport of carbon precursors (*e.g*., ADPG, G6P), which are finally stored in the endosperm of seeds. Therefore, the modified synthesis of endosperm starch can be achieved at least by the following pathways (Table 1), including (i) accelerating sucrose transport through leaf-phloem-seed; (ii) promoting the conversion of sucrose to UDPG in endosperm cells; (iii) promoting UDPG to enter amyloplasts in endosperm cells. Some starch-mediated strategies are summarized here for further applications of quality improvement in crop breeding.

**Table 1:** Strategies for grain improvement through starch biosynthesis.

Pathway	Strategy	Effectiveness
ATP supply	Down-regulated ANK	Increased ADPG, UDPG and starch contents
Sucrose synthesis	Over-expressed SUT1	Increased starch content
Sucrose → UDPG	Enhanced SuSy activity	Increased ADPG, UDPG and starch contents
UDPG → ADPG	Mutated Brittle1	Increased lipid content, but decreased protein and amylopectin contents
	Enhanced AGP activity	Increased starch content
	Over-expressed AGPase	Increased starch content
ADPG → Starch	Over-expressed GBSSI/Wx	Increased GC and AC
	Over-expressed SBE/ISA3	Increased GC and GT
	Over-expressed SSII	Increased GC, GT and GT
	Over-expressed SSIV	Change starch structure
	Down-regulated AMY	Increased amylopectin contents
	Over-expressed PPDK	Increased starch content, but decreased protein content
	Down-regulated Pho	Increased starch content

#### Promoted conversion from sucrose to starch

Although more enhanced transportation would benefit starch biosynthesis, overexpressed SUTs, one of the major sucrose transporters, could not significantly increase the starch content (Table 1). Nonetheless, it is feasible to promote the conversion to starch from sucrose that has entered the storage organs. Promoted conversion of sucrose to starch could effectively increase the starch content through the enhanced activity of SuSy. Overexpressed SuSy would significantly increase the contents of UDPG, ADPG, and starch (Bowsher et al., 2007; Li et al., 2013; Patron et al., 2004), and lead to higher AGP activity and higher amylopectin/amylose ratio (Asano et al., 2002; Cho et al., 2011). Therefore, it is feasible to increase the starch content by expressing SuSy into plastids to produce more ADPG. One possible explanation is that the SuSy–AGP–ADPG transporter-mediated starch synthesis and amylopectin or SP-regulated degradation reached a balance (Kazuaki, Kanako & Kazufumi, 2010; Li et al., 2013; Lee et al., 2016; Davies et al., 2003). Besides, SuSy competed with acid invertase to substrate sucrose and reached a balance to regulate the starch content (Baroja-Fernández et al., 2009; Murayama & Handa, 2007; Jia et al., 2008). This is supposed to be another reasonable mechanism. Therefore, the promoted conversion to starch from sucrose in the storage organs is feasible and seems to be subjected to the concentration balance of sucrose and starch (Table 1).

#### Increased supply of ATP

The supply of ATP is essential for storage starch biosynthesis; thus, the storage starch could be enhanced through the increased supply of ATP. For example, downregulated expression of plastidial adenylate kinase, an enzyme that catalyzes ATP to ADP and AMP, would increase the supply of ATP in amyloid and double starch content (Table 1). This may be related to the weakened competition between adenylate kinase and AGP to increase the ATP pool (Fernandez et al., 2017).

#### Promoted ADPG transport

As shown above, the downregulation of genes encoding plastidial adenylate kinase could increase the content of ADPG, which was one of the key precursors in starch synthesis. Besides increased synthesis, the enhanced transport of cytosolic ADPG to amyloid appeared to be another reasonable pathway to increase ADPG (Table 1). The enhanced expression of BT1 protein could promote the transport of cytosolic ADPG to amyloid, thereby increasing the endosperm’s starch content (Patron et al., 2004; Bowsher et al., 2007; Kirchberger et al., 2007; Li et al., 2017b). However, the *bt1* mutant had abnormal growth and infertility, which was not only related to decreased ADPG transport activity and starch deficiency in the endosperm amyloid (Kirchberger et al., 2007) but may also be related to some processes in the mitochondria (Bahaji et al., 2011). Nonetheless, due to the white heart endosperm and the decreased amylose content, the *bt1* mutant can serve as one of the floury materials in special food fields.

#### Enhanced activity of AGPase

As an important rate-limiting enzyme, the enhanced activity of AGPase would greatly increase the starch content (Table 1). Two ways were proposed to apply this strategy in seed improvement (Wang et al., 2013; Peng et al., 2014). One was the heterologous expression of the *E. coli glgC* gene in plants to produce AGP isomerase, which could significantly enhance AGP activity in seeds (Nagai et al., 2009). In contrast, another was the heterologous expression of the AGP large subunit encoding *SH2* gene (Pérez-Ruiz et al., 2006) and the small subunit encoding *BT2* gene (Pérez-Ruiz et al., 2006) in rice, which could significantly enhance AGPase activity and increase starch content. Thus, ways to enhance the activity of AGPase in seeds mainly focused on the heterologous expression of AGP isomerase. Therefore, it appears to be infeasible in breeding applications at present, given the legal limitations.

#### Regulated activity of starch synthase

Undoubtedly, starch biosynthetic enzymes’ regulation would greatly influence the biosynthesis of storage starch in seeds and mediate the grain quality, especially for eating and cooking qualities (ECQs). In rice, overexpressed GBSSI/Wx affected amylose content (AC) and gel consistency (GC) but had fewer effects on gelatinization temperature (GT) (Hanashiro et al., 2008). However, the overexpression of SSII affected AC, GC, and GT (Tian et al., 2009; Lin et al., 2013). Besides, the overexpression of ISA and SBE3 was prone to affect GC and GT (Yun, Takayuki & Yasushi, 2011). In addition to improving grain quality, the overexpression of some starch synthases would preferentially increase the starch content, *e.g*., SSIV (Guo et al., 2017; Gámez-Arjona et al., 2011).

Besides, modifications of starch biosynthetic genes could be a feasible pathway to improve grain quality and yields (Table 1). Recently, the editing of *ISA1 via* CRISPR/CRISPR-associated endonuclease 9 (CRISPR/Cas9) system has affected GT and starch chain length distribution during endosperm development, which have potential implications for quality improvement in rice (Chao et al., 2019). Moreover, repression of SSI by RNA interference (RNAi) could greatly affect starch biosynthesis and amylopectin chain distribution in rice under high temperature (Zhao et al., 2019). The suppression of *α*-amylase genes could also improve seed quality in rice under high temperature (Hakata et al., 2012).

#### Prevented degradation of starch

A network balance between starch synthesis and degradation resulted in enrichment of starch in endosperm. Therefore, it is feasible to increase starch content *via* preventing starch degradation (Bahaji et al., 2014; Fujita et al., 2009; Li et al., 2013). Alpha-amylase (Hakata et al., 2012; Seung et al., 2013) and the starch phosphorylation-related enzyme GWD (Ral et al., 2012) performed important regulatory roles in the degradation of endosperm starch. Under high temperature, the downregulated expression of alpha-amylase encoding genes would increase starch content (Hakata et al., 2012). Although the downregulated expression of GWD also increased starch content, it did not display the matched traits of dry weight, tiller number, and effective branch number of rice (Ral et al., 2012; Koumoto et al., 2013). Thus, it seems to be infeasible to produce varieties of high-quality through the single regulation of starch degradation enzymes, but indeed, it also provides a way to improve the contents of starch (Table 1).

#### Elevated contents of starch in leaves

Although leaf starch synthesis did not directly affect grain yield or quality as endosperm starch synthesis did, the biotechnological application could also be an effective way to improve crop starch content by altering or modifying related enzyme activities. The overexpression of OsCRCT, a CO_2_-Responsive CONSTANS, CONSTANS-like and Time of Chlorophyll a/b Binding Protein1 (CCT) Protein (CRCT), would significantly increase the starch content in the phloem of the leaf blade and leaf sheath during vegetative stages, which was supposed to be an alternative or potential approach to improve yields of food and biofuel (Morita et al., 2015). Recently, in maize, plants’ tolerance to high temperature has been realized through the modification of 6-phosphogluconate dehydrogenase (PGD) and originally plastid-localized enzymes (Ribeiro et al., 2020). PGD3 showed thermal stability in amyloplasts by fusing the chloroplast peptide encoding *Waxy1* into the open coding reading frame of PGD1 and PGD2, thus significantly improving plants’ heat resistance and yield in maize (Ribeiro et al., 2020). All these suggested the potential application of leaf starch biosynthetic genes to improve grain quality and yield (Table 1).

#### Breeding of high-amylose cereal varieties

Resistant starch (RS) is the sum of starch that could not digest and absorb by the small intestine (Reddy, Suriya & Haripriya, 2013), and its consumption can modulate postprandial metabolic responses (MacNeil et al., 2013). So, it possesses potential values for special populations, *i.e*., diabetics. The formation of RS largely depends on *GBSSI*/*W. expression*. SSIIIa regulates the formation of RS through the high expression of *Wx* gene, while its mutation would greatly suppress the *Wx* expression to produce resistant starch and amylose–lipid complexes (Ryoo et al., 2007; Hanashiro et al., 2008; Li et al., 2014b; Zhou et al., 2016). Other functioning enzymes in the formation of RS consist of pullulanase (Long et al., 2018) and amylase (Hakata et al., 2012). Recently, maize high-amylose lines with AC of more than 55% showed decreased expression of *BEIIb* and *SSIIIa* but the enhanced expression of ISA2, which was prone to extend amylopectin chains but restrain the length of short amylose chains (Zhong et al., 2020). Interestingly, we recently also found that the mutation of one plastid genes, *OsGUN4*, caused the high contents of amylose in rice (Li et al., 2021), which may be largely associated with the upregulated expression of *GBSSI*. Therefore, the formation of RS or high-amylose appears to be more relevant to the balance of *GBSSI* and other starch biosynthetic genes, *i.e*., *SSIIIa*, *BEIIb*, and *PUL*/*ISA*.

## Conclusion

Starch biosynthesis not only plays a critical role in the formation of grain yield and quality in cereal crops but also involves the coordination of different biological processes and various organs. These processes include sucrose synthesis and transport in source organs (such as leaves), sucrose and energy delivery from source organs throughout the vascular system (phloem) to sink organs (endosperm), conversion from sucrose to ADPG in the endosperm, and formation of amylopectin- and amylose-starch in the endosperm. The target-oriented improvement on yield and grain quality in cereal crops would optimize the current demand for starch to meet the living standard and be beneficial to overcome the problems in the availability of arable land by using the known mechanisms of starch biosynthesis.

## Supplemental Information

10.7717/peerj.12678/supp-1 Supplemental Information 1 Factors involved in starch biosynthesis.

## Additional Information and Declarations

### Data Availability

The following information was supplied regarding data availability: The factors involved in starch biosynthesis are available in the Supplementary File.
