# Supplementary material for: A review of starch biosynthesis in cereal crops and its potential breeding applications in rice (Oryza Sativa L.)
Source: PeerJ. 2021 Dec 22;9:e12678. doi: 10.7717/peerj.12678 (PMC8710062; doi:10.7717/peerj.12678)
Supplement: Supplemental Information 1 [file peerj-09-12678-s001.doc]

**Table S1** Factors involved in starch biosynthesis

| **Symbol** | **Encodes** | **Functions** | **Refs** |
| --- | --- | --- | --- |
| **Sucrose synthesis** | |  |  |
| FBA | Fructose-1,6-bisphosphate aldolase | Regulation of AGPase activity | maize (Guo et al., 2012) |
| FBP | Fructose 1,6-bisphosphatase | Catalyzes the first irreversible reaction from fructose-1,6-bisphosphate to fructose-6-phosphate and inorganic phosphate and plays an important regulatory role in sucrose biosynthesis and metabolism. | rice (Koumoto et al., 2013) |
| UGP | UDP-glucose pyrophosphorylase | The committed enzyme for the first step of sulfolipid biosynthesis | rice (Mu et al., 2009; Wang et al., 2020) |
| SPS | Sucrose phosphate synthase | Involved in the supply of solanesyl diphosphate for ubiquinone-9 biosynthesis in mitochondria. Farnesyl diphosphate is the preferred substrate. | rice (Kazuaki et al., 2010) |
| NIN3 | Alkaline/neutral invertase | Cleaves sucrose into glucose and fructose. | rice (Murayama et al., 2007; Jia et al., 2008) |
| SUTs | Sucrose transporters | Sucrose loading into the phloem in source and sink tissues | maize (Baker et al., 2016); rice (Sun et al., 2010); wheat (Aoki et al., 2004) |
| GIF1 | Cell-wall invertase/Grain incomplete filling 1 | Carbon partitioning during early grain filling | maize (Zhang et al., 2018) |
| A/N-inv | invertases | For the inverses of alkaline/neutral | rice (Murayama et al., 2007) |
| pPGM | Plastidic phosphoglucomutase | Starch synthesis in rice pollen | rice (Lee et al., 2016); wheat (Davies et al., 2003) |
| cPGM | Cytosolic phosphoglucomutase | Required for gametophyte development | maize (Pan et al., 1990) |
| **ADPG synthesis** | |  |  |
| AGPL1 | ADP-glucose pyrophosphorylase large subunit 1 | ADPG synthesis | rice (Peng et al., 2014) |
| AGPL2/GIF2 | ADP-glucose pyrophosphorylase large subunit 2/Grain incomplete filling 2 | ADPG synthesis | rice (Lee et al., 2007; Wei et al., 2017) |
| AGPL3 | ADP-glucose pyrophosphorylase large subunit 3 | ADPG synthesis | rice (Lee et al., 2007) |
| pAGPL4 | Plastidic large subunit of ADP-glucose pyrophosphorylase (AGP) | ADPG synthesis in pollen | rice (Lee et al., 2007) |
| AGPS1/ BT2 | Brittle 2/ADP-glucose pyrophosphorylase small subunit 1 | ADPG synthesis | rice (Lee et al., 2007) |
| AGPS2 | ADP-glucose pyrophosphorylase small subunit 2 | ADPG synthesis | rice (Lee et al., 2007) |
| **Starch synthesis** | | | |
| GBSSI/Wx1 | Granule-bound starch synthase I | For extra-long unit chains of amylopectin | rice (Hanashiro et al., 2008) |
| GBSSII | Granule-bound starch synthase II | For amylose synthesis | Rice (Chen et al., 2018) |
| SSI | DP<10, Soluble starch synthase | DP<10, soluble starch synthase | maize (Commuri et al., 2010); rice (Fujita et al., 2006) |
| SSIIa | Soluble starch synthase IIA | elongates chains to around 13–18 Glc units | rice (Jiang et al., 2004) |
| SSIIb | Soluble starch synthase IIB | elongates chains to around 13–18 Glc units | rice (Hirose et al., 2004) |
| SSIIIa/ FLO5 | Soluble starch synthase IIIA | synthesize long, cluster-spanning amylopectin chains | rice (Fujita et al., 2007; Ryoo et al., 2007) |
| SSIIIb | Soluble starch synthase IIIB | synthesize long, cluster-spanning amylopectin chains | rice (Li et al., 2014) |
| SSIVa | Soluble starch synthase IVA | Starch granule initiation | rice (Dian et al., 2005) |
| SSIVb | Starch synthase IV | Coordination of starch granule formation with chloroplast division | Wheat (Guo et al., 2017) |
| BEI | Starch branching enzyme I | Production of inner branches | maize (Blauth et al., 2002); rice (Satoh et al., 2003); wheat (Rahman et al., 1997) |
| BEIIa | Starch branching enzyme II a | Endosperm starch synthesis and compound granule formation | wheat (Rahman et al., 1997) |
| BEIIb | Starch branching enzyme II b | Formation of amylopectin A chains | rice (Nishi et al., 2001); wheat (Regina et al., 2005); maize (Liu et al., 2012) |
| BEIII | Starch branching enzyme III | Formation of amylopectin chains | wheat (Kang et al., 2013) |
| BEIVa | Starch branching enzyme IV | Transfers part of a linear glucan chain to another chain, forming an α-1,6-linkage | rice (Mizuno et al., 2001) |
| ISA1 | Isoamylase-typ enzyme 1e debranching | Debranch phytoglycogen and amylopectin | maize (Dinges et al., 2003) |
| ISA2 | Isoamylase-type starch debranching enzyme ISO2 | For the activity of ISA heteromeric enzymes | maize (Lin et al., 2013); rice (Utsumi et al., 2011) |
| ISA3 | Isoamylase-type debranching enzyme 3 | Debranch phytoglycogen and amylopectin; Granule initiation | rice (Yun et al., 2011) |
| PUL | Pullulanase-type debranching enzyme | Starch degradation during kernel gemination and starch biosynthesis; Essential for starch degradation in leaves | rice (Fujita et al., 2009); maize (Wu et al., 2002; Dinges et al., 2003); |
| Pho 1/Pho 2 | Plastidial α-glucan phosphorylase | Affects the synthesis and structure of starch in the endosperm | rice (Secco et al., 2010; Satoh et al., 2008) |
| SuSy | Sucrose synthase | Produce UDPG | rice (Asano et al., 2002; Cho et al., 2011) |
| UGP1/FLO8 | UDP-glucose pyrophosphorylase 1 | For calluose deposition during pollen mother cell and meiosis stages and for seed carbohydrate metabolism | maize (Mechin et al., 2007); rice (Long et al., 2017) |
| UGP2 | UDP-glucose pyrophosphorylase 2 | Plays a key role during pollen maturation, especially for starch accumulation | rice (Mu et al., 2009) |
| BT1 | Brittle 1, ADP-glucose/ADP transporter | Transport ADPG in counter exchange with ADP | rice (Li et al., 2017c); maize (Kirchberger et al., 2007); barley (Patron et al., 2004); wheat (Bowsheret al., 2007) |
| SnRK1 | Sucrose-nonfermentation1-related protein kinase1 | Role in sugar and ABA signaling | rice (Cho et al., 2012) |
| Tre6P | Trehalose 6-phosphate | Coordinate organic and amino acid metabolism | maize (Bledsoe et al., 2017) |
| NTRC | Plastidial NADP-thioredoxin reductase | Involved in regulation of SS3 and SS4 | rice (Pérez-Ruiz et al., 2006) |
| FLO6 | Floury 6, a CBM48 domain-containing protein | Involved in compound granule formation and starch synthesis | rice (Peng et al., 2014) |
| PPDK/FLO4 | Pyruvate orthophosphate dikinase | Involved in endosperm starch synthesis, compound granule formation and grain filling | rice (Cai et al., 2018; Kang et al., 2005); maize (Mechin et al., 2007) |
| **Transcription Factors (TFs)** | |  |  |
| bZIP58 | Basic leucine zipper transcription factor 58 | Regulate the expression of *AGPL*, *Wx*, *SS2a*, *BE1*, *BE2b* and *ISA2* | rice (Wang et al., 2013) |
| bZIP91 | Basic leucine zipper transcription factor 91 | Regulate genes of starch biosynthesis | maize (Chen et al., 2016) |
| NAC36 | Transcription factor | Co-expression of starch synthetic genes | maize (Zhang et al., 2014) |
| EREB156 | AP2/EREBP transcription factor | Positively modulates starch biosynthetic gene *SSIIIa* via the synergistic effect of sucrose and ABA | maize (Huang et al., 2016) |
| FLO2 | Floury endosperm 2 | Regulation of grain size and starch quality | rice (She et al., 2010) |
| FLO7 | Floury endosperm 7 | A regulator for starch synthesis and amyloplast development during endosperm development | rice (Zhang et al., 2016b) |
| GRAS20 | GRAS TF | Regulate genes of starch biosynthesis | rice (Cai et al., 2017); maize (Li et al., 2014) |
| RSR1 | APETALA2/ethylene-responsive element binding protein family TF | Regulate genes of starch biosynthesis | rice (Fu and Xue, 2010) |
| SUSIBA2 | WRKY TF | Participates in sugar signaling by binding to the sugar-responsive elements of the ISO1 promoter | barely (Sun et al., 2003) |
| O2/PBF | An endosperm-specific TF | Regulate the protein and starch synthesis by PPDK | maize (Zhang et al., 2016a) |
